# CARD 2020: antibiotic resistome surveillance with the comprehensive antibiotic resistance database

**DOI:** 10.1093/nar/gkz935

**Published:** 2019-10-29

**Authors:** Brian P Alcock, Amogelang R Raphenya, Tammy T Y Lau, Kara K Tsang, Mégane Bouchard, Arman Edalatmand, William Huynh, Anna-Lisa V Nguyen, Annie A Cheng, Sihan Liu, Sally Y Min, Anatoly Miroshnichenko, Hiu-Ki Tran, Rafik E Werfalli, Jalees A Nasir, Martins Oloni, David J Speicher, Alexandra Florescu, Bhavya Singh, Mateusz Faltyn, Anastasia Hernandez-Koutoucheva, Arjun N Sharma, Emily Bordeleau, Andrew C Pawlowski, Haley L Zubyk, Damion Dooley, Emma Griffiths, Finlay Maguire, Geoff L Winsor, Robert G Beiko, Fiona S L Brinkman, William W L Hsiao, Gary V Domselaar, Andrew G McArthur

**Affiliations:** 1 David Braley Centre for Antibiotic Discovery, McMaster University, Hamilton, Ontario, L8S 4K1, Canada; 2 M.G. DeGroote Institute for Infectious Disease Research, McMaster University, Hamilton, Ontario, L8S 4K1, Canada; 3 Department of Biochemistry and Biomedical Science, McMaster University, Hamilton, Ontario, L8S 4K1, Canada; 4 Bachelor of Health Sciences Program, McMaster University, Hamilton, Ontario, L8S 4K1, Canada; 5 Honours Biology Program, McMaster University, Hamilton, Ontario, L8S 4K1, Canada; 6 Bachelor of Arts & Science Program, McMaster University, Hamilton, Ontario, L8S 4K1, Canada; 7 Center for Genome Sciences, National Autonomous University of Mexico, Cuernavaca, Morelos 62210, Mexico; 8 Department of Genetics, Harvard Medical School, Harvard University, Boston, MA 02115, USA; 9 Department of Pathology and Laboratory Medicine, University of British Columbia, Vancouver, V6T 2B5, British Columbia, Canada; 10 Department of Molecular Biology and Biochemistry, Simon Fraser University, Burnaby, British Columbia, V5A 1S6, Canada; 11 Faculty of Computer Science, Dalhousie University, Halifax, Nova Scotia, B3H 1W5, Canada; 12 British Columbia Centre for Disease Control Public Health Laboratory, Vancouver, British Columbia, V5Z 4R4, Canada; 13 National Microbiology Laboratory, Public Health Agency of Canada, Winnipeg, Manitoba, R3E 3R2, Canada; 14 Department of Medical Microbiology and Infectious Diseases, Max Rady College of Medicine, University of Manitoba, Winnipeg, Manitoba, R3E 0J9, Canada

## Abstract

The Comprehensive Antibiotic Resistance Database (CARD; https://card.mcmaster.ca) is a curated resource providing reference DNA and protein sequences, detection models and bioinformatics tools on the molecular basis of bacterial antimicrobial resistance (AMR). CARD focuses on providing high-quality reference data and molecular sequences within a controlled vocabulary, the Antibiotic Resistance Ontology (ARO), designed by the CARD biocuration team to integrate with software development efforts for resistome analysis and prediction, such as CARD’s Resistance Gene Identifier (RGI) software. Since 2017, CARD has expanded through extensive curation of reference sequences, revision of the ontological structure, curation of over 500 new AMR detection models, development of a new classification paradigm and expansion of analytical tools. Most notably, a new Resistomes & Variants module provides analysis and statistical summary of *in silico* predicted resistance variants from 82 pathogens and over 100 000 genomes. By adding these resistance variants to CARD, we are able to summarize predicted resistance using the information included in CARD, identify trends in AMR mobility and determine previously undescribed and novel resistance variants. Here, we describe updates and recent expansions to CARD and its biocuration process, including new resources for community biocuration of AMR molecular reference data.

## INTRODUCTION

In the century since Alexander Fleming isolated penicillin (1,2) and later warned about antibiotic resistance (3), the world of clinical therapeutics has been transformed by antibiotic discovery and their widespread use (4). However, antibiotic misuse and poor stewardship have turned antimicrobial resistance (AMR) into a global health crisis, exacerbated by a withered antibiotic discovery pipeline (5). This has spurred a collaborative global effort to combat AMR, improve antimicrobial stewardship, and advance surveillance of resistance determinants (6–9). With the increasing use of genome sequencing as a surveillance tool for AMR molecular epidemiology (10,11), as well as the targeting of specific AMR genes by novel adjuvants (12), databases and clear nomenclature for AMR gene families is critical. Given the severity of the AMR crisis and the next-generation sequencing revolution, it is no surprise that there is a large diversity of AMR databases and software tools available (10,13). Many of these are highly focused on, for example, metagenomics of environmental AMR (14), profiling for AMR conferring mutations in *Mycobacterium tuberculosis* (15) or collation of AMR-associated transposable elements (16). Others re-package the content of other AMR databases to provide an alternative database (17), tool (18) or statistical model (19). A small number are primary AMR databases that curate information from the scientific literature into their database to support sequence analysis and knowledge integration. Most notable of these primary AMR databases are ARG-ANNOT (20), ResFinder (21) and increasingly the National Center for Biotechnology Information (NCBI) Pathogen Detection Reference Gene catalog (22). We previously introduced the Comprehensive Antibiotic Resistance Database (CARD; card.mcmaster.ca; (23,24)), a primary bacterial AMR knowledge resource and database which provides genotype analysis and phenotype prediction from curated publications and sequences. In our 2017 update (24), we detailed the reorganization of CARD around a new Model Ontology, which allowed AMR sequence and mutation reference data to be organized by the underlying specific mechanisms of resistance, with subsequent improvements in CARD’s Resistance Gene Identifier (RGI) algorithms. We here describe (i) the expanded biocuration of reference sequences and mutation data in CARD, (ii) expansion of CARD’s Antibiotic Resistance Ontology (ARO) to include terms for harmonization of AMR phenotypic assays, (iii) *in silico* surveillance of pathogen resistomes and sequence variants, (iv) new tools for classification of reference data and genome annotation results and (v) new efforts toward community biocuration of AMR molecular reference data.

## EXPANSION OF CARD

### Current state of CARD and the ARO

CARD integrates molecular biology, biochemistry and bioinformatics within an ontological framework to produce a database that is both functional and practical for clinicians, researchers, industry and public health agencies. The primary objective of CARD is to harmonize and standardize, through expert human curation, AMR molecular sequence knowledge to produce a reliable and trustworthy central database of sequences and mutations known to confer AMR. All curated data within CARD are organized using controlled vocabularies (i.e. ontologies), with four such ontologies being central to its operation: the ARO, the CARD Model Ontology (MO), the CARD Relations Ontology (RO; an augmented subset of the Open Biological and Biomedical Ontology (OBO) Relations Ontology) (http://purl.obolibrary.org/obo/ro) and NCBITaxon (a curated subset of the NCBI Organismal Taxonomy Ontology (22)) (http://purl.obolibrary.org/obo/ncbitaxon). The ARO is the primary ontology in CARD as it includes detailed descriptions of the molecular basis for antibiotic resistance, encompassing known AMR determinants (i.e. acquired resistance genes, resistant mutations of housekeeping genes, efflux overexpression, etc.), drug targets, antibiotic molecules and drug classes, and the molecular mechanisms of resistance. The ARO is organized into three major branches: Determinant of Antibiotic Resistance (ARO:3000000), Antibiotic Molecule (ARO:1000003) and Mechanism of Antibiotic Resistance (ARO:1000002). Each resistance determinant described by the ARO (e.g. an individual β-lactamase) must include an ontological connection to each of these three branches. Additional, minor ARO branches detail other aspects of AMR: Antibiotic Target (ARO:3000708), for describing antibiotic-sensitive wild-type bacterial components; Antibiotic Biosynthesis (ARO:3000082), for describing *in vivo* antibiotic synthesis by bacterial cells or communities; and, Resistance-Modifying Agents (ARO:0000076), for describing antibiotic adjuvants, inhibitors of resistance enzymes, and antibiotic potentiators which help restore a susceptible phenotype. Since our previous update and in collaboration with the Genomic Epidemiology Ontology (GenEpiO.org), we have added a new AMR Phenotype Terminology branch (ARO:3000045) to the ARO containing 133 terms describing clinical AMR phenotypes, laboratory microbial susceptibility testing and testing reference standards. Overall, each entity in ARO uses semantic relationships within and between these branches to provide the full biochemical context for each AMR determinant, some of which have been updated (Table 1). Additionally, CARD has recently launched draft ontologies for both virulence (VIRO; 701 ontology terms) and mobile genetic elements (MOBIO; 283 ontology terms), which are in active development.

**Table 1.** Ontological relationships used by CARD within the Antibiotic Resistance Ontology (ARO)

Relationship Label	Accession	Description^4^
*is_a*	n/a	An axiomatic relationship wherein the subject class *A* is a subclass of class *B*
*part_of*	BFO^1^:0000050	A relationship wherein a subject class *A* is but a part of class *B*
*has_part*	BFO:0000051	A relationship wherein a subject class *A* has a part class *B* (inverse of *part_of*)
*participates_in*	RO^2^:0000056	A relationship between continuant *A* and process *B* wherein *A* is somehow involved in *B*
*regulates*	RO:0002211	A relationships wherein the subject class *A* regulates the activity of class *B*
*derives_from*	RO:0001000	A relationship between class *A* and class *B* wherein *B* inherits many properties from *A*
*evolutionary_variant_of*	RO:0002321	A relationship wherein gene or protein *A* is a paralogous or orthologous variant of gene or protein *B*
*confers_resistance_to_drug_class*	Pending^3^	A relationship wherein the subject class *A* confers or contributes to antibiotic resistance to drug class *B* (formerly *confers_resistance_to*)
*confers_resistance_to_antibiotic*	Pending	A relationship wherein the subject class *A* confers or contributes to antibiotic resistance to antibiotic *B* (formerly *confers_resistance_to_drug*)
*targeted_by*	Pending	A relationship wherein molecule *A* is targeted by drug class *B*
*targeted_by_antibiotic*	Pending	A relationship wherein molecule *A* is targeted by antibiotic *B* (formerly *targeted_by_drug*)

^1^Basic Formal Ontology (http://purl.obolibrary.org/obo/bfo).

^2^Relations Ontology (http://purl.obolibrary.org/obo/ro).

^3^Custom relationships for CARD used by ARO but not yet included in the Relations Ontology.

^4^Paraphrased from source.

CARD curation occurs continuously, with monthly updates released by a team of biocurators. CARD curation involves both a descriptive component (i.e. an ontology term) and a functional component (i.e. AMR detection models with associated reference sequences). The curation process primarily involves regular review of the available scientific literature, as described in detail below, to determine applicable additions and modifications. Enforced curation guidelines provide the necessary context to ensure proper hierarchical classification, defined semantic relationships and data standardization. For example, when a new resistance determinant is identified, a biocurator places it within the ARO with the appropriate ontological relationships to indicate the AMR gene family, resistance mechanism and observed drug-class resistance. The biocuration team additionally annotates each ARO term with supplemental information from external references, including relevant publications (via NCBI PubMed (22)), chemical structures (for antibiotics in particular, via NCBI PubChem (25)) or protein structure via the Protein DataBank (rcsb.org; 26)). At last, ARO terms for AMR determinants are paired with an AMR detection model, which includes the nucleotide and peptide sequence retrieved from NCBI GenBank and any additional parameters needed for prediction of the determinant from raw DNA sequence (outlined below). Curation is sometimes supplemented with *de novo* analyses, often to resolve problematic nomenclature, as we recently performed for trimethoprim resistant dihydrofolate (dfr) reductases .

Overall, **CARD’s primary curation paradigm** is as follows: to be included in CARD an AMR determinant must be described in a peer-reviewed scientific publication, with its DNA sequence available in GenBank, including clear experimental evidence of elevated minimum inhibitory concentration (MIC) over controls. AMR genes predicted by *in silico* methods, but not experimentally characterized, are not included in CARD’s primary curation. Yet, our data harmonization efforts in 2019 that involved a comparison of ResFinder (21), ARG-ANNOT (20) and NCBI’s catalog of β-lactamase alleles (27), revealed a large number of historical β-lactamases without associated peer-reviewed publication. As β-lactamases comprise nearly a third of ARO terms in CARD, that convention leads to each β-lactamase sequence variant being given a new name in the literature and missing β-lactamase reference sequences in CARD resulted in annotation imprecision by RGI and notable content differences between CARD and other databases, CARD now includes β-lactamase reference sequences and names even if they lack published experimental evidence of elevated MIC. This back-curation of older β-lactamase sequences is ongoing. The antibiotic molecule branch is another area of active curation: while 80% (278 out of 342) of ARO antibiotic terms are harmonized with the NCBI BioSample database (28), CARD curation rules require each antibiotic in the ARO to be cross-referenced to a PubChem ID (PCID), which some molecules lack. As such, current curation efforts aim to complete ARO harmonization by including other structural databases such as SciFinder (29), DrugBank ((30) and ChEBI (31).

In summary, as of September 2019 the size of the ARO has grown considerably, from 3567 (24) to 4336 ontology terms, covering resistance mechanisms from 2923 AMR determinants (plus an additional 1304 resistance variant mutations), all supported by 2648 curated publications. The increased number of curated mutations is in part due to new CARD curation rules allowing inclusion of mutations discovered by laboratory selection experiments, in addition to mutations discovered and characterized from clinical, agricultural or environmental isolates. This is a new level of biocuration in CARD and the distinction is clearly labeled at the website and in provided download files. Additionally, as of the CARD 3.0.3 release version (July 2019) we now detect microbial name changes at NCBI not incorporated into CARD and subsequently update CARD to reflect the latest pathogen taxonomy, e.g. *Enterobacter aerogenes* renamed to *Klebsiella aerogenes*.

### Simplifying interpretation with ARO classifications

With over 4300 terms, the ARO provides a powerful framework for organization and interpretation of the molecular basis of AMR. As a graph, it has proven essential for accurate biocuration of AMR, visual presentation of data on the CARD website, automated error checking and as a data framework for bioinformatics software such as RGI. Yet, its complexity does not lend itself to easy human interpretation, e.g. the NDM-1 β-lactamase (ARO:3000589) has relationships to 28 ontology terms within the ARO, including *confers_resistance_to_antibiotic* ertapenem, the carbapenem β-lactams, the category class B (metallo-) beta-lactamase and hydrolysis of antibiotic conferring resistance. To address this issue, we have added a new ARO classification tagging paradigm, where our expert curators manually ‘tag’ certain terms in the ARO as particularly informative for interpretation. We designed seven types of classification tags: four primary tags used to index and classify genome or metagenome annotation results (AMR Gene Family, Drug Class, Resistance Mechanism, Antibiotic) and three secondary tags to track adjuvants or the complexities of antimicrobial efflux (Efflux Component, Efflux Regulator, Adjuvant) (Table 2). For example, the primary ARO classification for NDM-1 β-lactamase includes the AMR Gene Family ‘NDM β-lactamase’ (ARO:3000057), Resistance Mechanism ‘antibiotic inactivation’ (ARO:0001004), and Drug Classes carbapenem (ARO:0000020), cephalosporin (ARO:0000032), cephamycin (ARO:0000044) and penam (ARO:3000008). NDM-1 also has primary Antibiotic ARO classifications for amoxicillin-clavulanic acid, ertapenem, imipenem and meropenem based on curated *confers_resistance_to_antibiotic* relationships. Overall, the ARO classification tags were chosen carefully based on the existing ARO hierarchies, sequence similarities, conventions in the scientific literature and compatibility with future database development.

**Table 2.** ARO classification tags used to drive biocuration and provide easy interpretation of genome annotations

Classification Tag	Requirement^1^	Annotated ARO Terms	ARO Example^2^
AMR Gene Family	Primary	304	NDM ß-lactamase (ARO:300057)
Drug Class	Primary	49	Aminoglycoside (ARO:0000016)
Resistance Mechanism	Primary	7	Antibiotic target replacement (ARO:0001002)
Antibiotic	Primary	308	Streptomycin (ARO:0000040)
Adjuvant	Secondary	8	Tazobactam (ARO:0000077)
Efflux Component	Secondary	1	Efflux pump complex or subunit (ARO:3000159)
Efflux Regulator	Secondary	1	Two-component regulatory system modulating efflux (ARO:3000451)

^1^Primary tags are required for all CARD AMR determinants where applicable; secondary tags apply only rarely and can be omitted at the curator's discretion.

^2^Example names are abbreviated, see ARO accession in CARD for the complete description.

**Figure 1.**
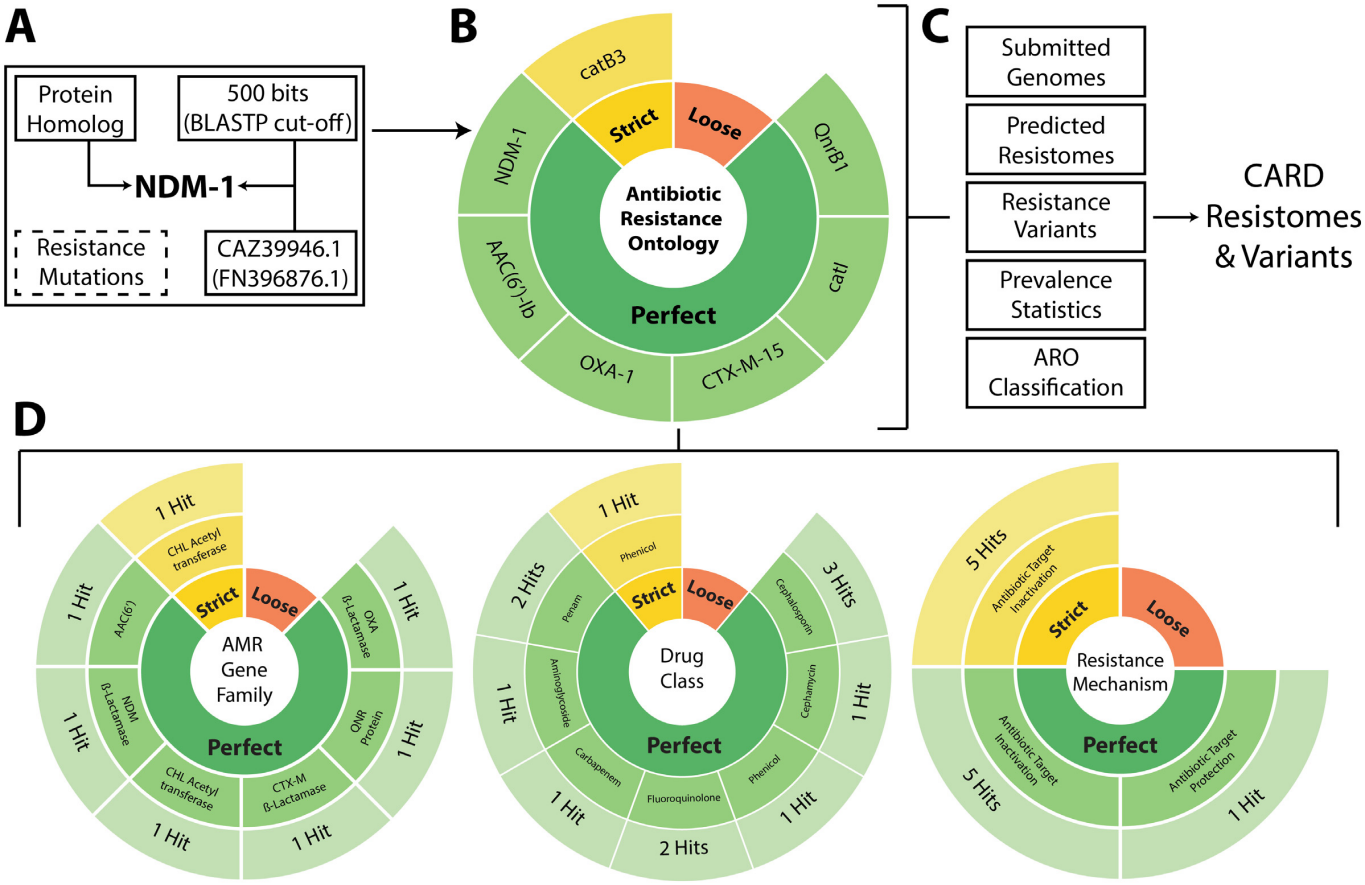
Overview of CARD’s Resistance Gene Identifier software and its role in generating the CARD Resistomes & Variants data. (**A**). CARD AMR detection models include a reference sequence, a curated BLAST(P/N) bit score cut-off, and, if applicable, mutations known to predict AMR. This example shows the model parameters curated for the metallo-ß-lactamase NDM-1 (NCBI GenBank accession CAZ39946.1, from *Klebsiella pneumoniae* plasmid pKpANDM-1 accession FN396876.1). (**B**). User-submitted queries are analyzed by RGI using detection models which generate an annotation organized by the Perfect, Strict and Loose (if selected) paradigm. Here, we show the predicted resistome and CARD generated visualizations identified from NCBI GenBank accession JN420336.1 (*Klebsiella pneumoniae* plasmid pNDM-MAR). (**C**). When performed across thousands of complete genome sequences, complete plasmid sequences and WGS assemblies for 82 pathogens, the resulting data is extracted and calculated to populate the CARD Resistomes & Variants module. (**D**). Illustration of ARO classification tagging for JN420336.1, allowing organization of RGI results by AMR Gene Family, Drug Class and Resistance Mechanism.

With addition of ARO classification tags, we have **expanded CARD’s curation paradigm** as follows: every curated AMR determinant must have an ontological path including each of the four primary ARO classification tags, i.e. the AMR Gene Family to which that determinant belongs, the Resistance Mechanism, the Drug Class(es) to which resistance is conferred, and the specific Antibiotic with a demonstrably elevated MIC. This tagging allows easy interpretation of resistome predictions (Figure 1). To date, 670 ARO terms have been tagged for ARO classification. Among primary tags, these include 304 AMR Gene Family tags, 49 Drug Class tags, 7 Resistance Mechanism tags and 308 Antibiotic tags. As a result, nearly all of the 2923 AMR detection models and 2890 reference sequences in CARD have ARO classification for AMR Gene Family, Drug Class and Resistance Mechanism (a minority are mid-curation). Many additionally have ARO classification for Antibiotic, yet curation of *confers_resistance_to_antibiotic* relationships is ongoing and incomplete as this is a new area of emphasis for CARD, with the goal of curating all published *confers_resistance_to_antibiotic* relationships, including reported MICs, by the end of 2020. We note that CARD’s new ARO classification paradigm is analogous to MEGARes’ (17) acyclic graph organization of AMR reference sequences, which powers the popular AMR++ metagenomics tool (17) and the recently reported Meta-MARC Hidden Markov Models (32). CARD and MEGARes will be collaborating in 2019–2020 to harmonize these efforts, allowing CARD curation updates to seamlessly pass to MEGARes, AMR++ and Meta-MARC.

### Ensuring comprehensive biocuration

While a large part of CARD’s value is expert, human biocuration of AMR sequence data and its relationship to antibiotics, with AMR publications in PubMed exceeding over 5000 per year for the last 10 years (based on PubMed MeSH records for ‘Drug Resistance, Microbial’) the task of keeping CARD both comprehensive and up-to-date is daunting. CARD addresses this problem using three approaches: *ad hoc* biocuration, pathogen AMR reviews and computer-assisted literature triage. *Ad hoc* biocuration involves addressing feedback from the AMR research community as well as literature discovered during quality-control (QC) checks or review of AMR gene nomenclature. Pathogen AMR review involves systematic review of the AMR literature for specific pathogens, with reviews completed in the last 2 years for *Acinetobacter baumannii*, *Chlamydia trachomatis*, *Clostridioides difficile*, *Escherichia coli*, *Mycoplasma genitalium*, *Neisseria gonorrhoeae* and *Pseudomonas aeruginosa*. Biocuration of *M. tuberculosis* AMR will be a major focus in 2020, including harmonization with ReSeqTB (33), as CARD currently has curation gaps for this pathogen. In 2017, we described the CARD*Shark text-mining algorithm (26) for computer-assisted literature triage, which we have expanded based on the new ARO Drug Class classification tags. CARD*Shark assigns priority scores to publications from a general PubMed Medical Subject Headings (MeSH) search based on relevance and assigns the results to a CARD biocurator for manual review.

### Expanded and higher resolution AMR detection models

AMR determinants (resistance gene sequences, variants or specific mutations) are associated with ARO terms and AMR detection models in CARD, thus providing the interpretive context (ARO), reference sequence data and bioinformatics parameters for prediction of AMR determinants from raw DNA sequence. The latter is described by CARD’s Model Ontology (MO, Supplementary Figure S1), which includes reference nucleotide and protein sequences, as well as additional search parameters including mutations conferring AMR (if applicable) and curated BLAST(P/N) (34,35) bit score cut-offs. The majority of CARD AMR determinants use either a protein homolog model (PHM, e.g. a β-lactamase) or a protein variant model (PVM, e.g. a mutation in gyrase subunit A conferring resistance to fluoroquinolone). PHMs predict AMR protein sequences from raw DNA sequence based on homology to a curated reference sequence, based on a curated BLAST bit score cut-off. PVMs perform a similar search, but include additional parameters for the detection of specific curated non-synonymous mutations or other genetic variants (i.e. INDELs, frameshifts) that differentiate between antibiotic-susceptible wild-type and antibiotic-resistant alleles. Since 2017, we have transitioned each detection model in CARD to curated BLAST bit score (*S′*) cut-offs, discontinuing use of less discriminatory BLAST expectation values (*E*). The chosen bit score cut-off reflects a human curated value that aims to differentiate between putative functional homologs and other similar proteins with different functions. Bit score cut-offs are selected based on values that perform this discrimination when the curated reference sequence is compared by BLAST against CARD itself and against GenBank's non-redundant database, with hand inspection to determine a value that correctly classifies matches as homologs of similar antimicrobial function (i.e. ≥ bit score cut-off) or similar proteins with different function or AMR Gene Family membership (i.e. <bit score cut-off). We had determined that the asymptotic nature of the BLAST expectation value (E) gave it very low discriminatory power between different β-lactamase gene families (nearly ⅓ of CARD’s content), but that the linear nature of the BLAST bit score (S′) allowed this level of discrimination.

CARD now also includes two additional model types, the rRNA gene variant model (RVM) and the protein overexpression model (POM). The RVM is functionally similar to the PVM, except it works for rRNA mutations and therefore uses a nucleotide reference sequence and a BLASTN bit score cut-off. The POM is also similar to the PVM, but predicts protein overexpression based on the presence of mutations often associated with regulatory proteins. POM reflects how certain proteins contributed to AMR with and without mutations and is most often applied to efflux complexes, where wild-type proteins result in low or basal expression, whereas key mutations result in overexpression and clinical resistance (36,37). Unlike RVMs, which report only antibiotic-resistant alleles, POMs report detection of wild-type efflux complexes known to act upon antibiotics at basal levels or mutant complexes with likely overexpression and clinical resistance. As of September 2019, 80 RVMs and 12 POMs have been added to CARD, joined by 2611 PHMs (+509 since 2017) and 156 PVMs (+64 since 2017). Overall, CARD’s 2923 AMR detection models are comprised of 2890 reference sequences and 1304 amino acid substitution mutations, in addition to many other AMR-associated mutations (INDELs, nonsense mutations, frameshift mutations, etc.).

### Resistance gene identifier version 5

Spring 2019 saw release of CARD’s RGI software version 5, which uses the integrated information in CARD to predict resistome for genomic and metagenomic data, either using CARD’s website or as a command-line tool. Briefly, RGI algorithmically predicts AMR genes and mutations from submitted genomes using a combination of open reading frame prediction with Prodigal (38), sequence alignment with BLAST (35) or DIAMOND (39), and curated resistance mutations included with the AMR detection model. A manuscript detailing RGI’s algorithms is in preparation, but a few improvements are worth noting as they reflect changes in CARD content. First, RGI now supports annotation of metagenomic reads in addition to the previously supported annotation of genome or genome assembly sequences. Metagenomics analysis (i.e. RGI *bwt*) uses Bowtie2 (40) or BWA (41) mapping of sequencing reads to CARD’s PHM reference sequences only, while annotation of genomes or assembly contigs predicts resistome using four of CARD’s AMR detection models: PHM, PVM, RVM and POM (note: RGI currently only scans for non-synonymous substitutions; not frameshifts, deletions or insertions). Both classify results using CARD’s new ARO classification tags (Figure 1). Metagenomics analysis uses standard read mapping statistics (MAPQ, depth of coverage, length of coverage, etc.) while annotation of genomes or assembly contigs retains RGI’s Perfect/Strict/Loose paradigm (24). The ‘Perfect’ algorithm detects AMR proteins with an exact (100%) match to a CARD reference sequence, while the ‘Strict’ algorithm is more flexible, allowing for variation from the CARD reference sequence as long as the sequence falls within the curated BLAST bit score cut-offs, and is useful for detecting previously unknown variants of AMR genes or antibiotic targets altered via mutation. The ‘Loose’ algorithm works outside of the detection model cut-offs to provide detection of new, emergent threats and more distant homologs of AMR genes, but will also catalog homologous sequences and spurious partial hits that may not have a role in AMR. Combined with phenotypic screening, the Loose algorithm potentiates novel AMR gene discovery and research.

### CARD resistomes, variants and prevalence

The AMR reference data included in CARD is derived exclusively from peer-reviewed publications, following CARD’s curation paradigm. Thus, CARD biocuration precludes putative AMR determinants or variants not validated by clinical or experimental data. To wit, CARD reference sequences do not include computationally predicted alleles lacking an experimental demonstration of elevated MIC over controls. Yet, assessment of sequence diversity is important for epidemiological investigations, evolutionary studies, mapping of metagenomic sequencing reads (42) and construction of Hidden Markov Models (32). To fill this gap in the available resources, we developed the new CARD module ‘CARD Resistomes & Variants’, a collection of computationally predicted resistome data (https://card.mcmaster.ca/genomes). To generate these data, we analyzed pathogen genomes with RGI to produce a predicted resistome for each, tracking allelic variation, ARO classification, and prevalence among pathogens, genomes, plasmids, and whole genome shotgun (WGS) assemblies. In total, CARD Resistomes & Variants includes *in silico* surveillance of 82 pathogens of public health and AMR relevance, including each pathogen from the World Health Organization's (WHO) Global Priority List of Antibiotic-Resistant Bacteria (9). For each of these pathogens, we retrieve all available NCBI RefSeq complete genome sequences, complete plasmid sequences, and WGS assemblies and predict resistomes using RGI and the CARD AMR detection models (Supplementary Table S1), retaining ‘Perfect’ and ‘Strict’ hits only (Figure 1). These results are used to generate a collection of sequence variants (i.e. AMR alleles), annotated resistomes, and AMR gene prevalence statistics, all organized by ARO classification tags and browsable or downloadable at the CARD website. For example, CARD Resistomes & Variants (September 2019) reports that the TEM-1 β-lactamase gene has 25 alleles among 26 different pathogens, including plasmid-borne copies found in *Enterobacter* spp., *E. coli*, *N. gonorrhoeae* and others, plus genomic incorporation in *A. baumannii*, *Haemophilus influenzae*, *Salmonella enterica*, and others. As of September 2019, CARD Resistomes & Variants includes 92,894 predicted alleles (55,994 encoded proteins) covering 1656 AMR detection models from 82 pathogens. CARD Resistomes & Variants are not included in CARD’s primary curation nor used as reference sequences, except that CARD’s RGI version 5 can optionally incorporate these data to increase reference sequence diversity for mapping of metagenomic reads, to provide epidemiological context for interpretation of metagenomic data, and to provide novel k-mer algorithms (i.e. signature sub-sequences) for pathogen-of-origin and plasmid-association predictions for AMR genes or metagenomic reads (manuscript in preparation, but see https://www.github.com/arpcard/rgi). To maintain a clear distinction between characterized AMR alleles and *in silico* predictions, these two forms of data are accessible on different parts of the CARD website and via separate download files.

### Schema and information technology

CARD uses the custom ‘Broad Street’ schema for storage and curation (24), named for the 1854 Broad Street cholera outbreak and pioneering epidemiological efforts of Dr John Snow (43). The schema now contains six modules: controlled vocabularies; AMR detection models; resistomes, variants & prevalence; publication; external reference; and, administrative. The schema and data are managed with PostgreSQL 9.5 and the public CARD website and curator tools are designed with the Laravel 5.2 PHP framework, PHP 7.0.22, Apache 2.4 and PostgreSQL 9.5. Additional statistics are generated with Biopython (44). The website, software, data and curation issue tracking are all version-controlled using GitLab CE version 11.5.0. The CARD website had over 1 million page views by over 100 000 users from September 2016 to September 2019, with 77.9% new visitors and 22.1% returning visitors. Usage was global: Asia 35.62%, Americas 32.53%, Europe 26.86%, Africa 2.67% and Oceania 2.11% (with 0.22% indeterminant). In the same time period, the CARD website hosted ∼45 000 BLAST analyses, ∼220 000 RGI analyses, ∼64 000 data file downloads, and ∼10,000 RGI software downloads.

### Updates, availability and community AMR curation

The CARD curation team continuously updates the database on a development server and prior to release, rigorous QC scripts are implemented to validate these data before porting it to the publicly available website. These QC steps verify the use of external identifiers, publication citations, AMR detection model parameters and imposed rules for the ontology structure. Any detected issues are resolved prior to release. After QC, the public CARD website (https://card.mcmaster.ca) is updated monthly (with a few exceptions) and provides tools for browsing and searching the ARO, AMR detection model parameters and reference sequences, CARD Resistomes & Variants (https://card.mcmaster.ca/genomes) data with Prevalence calculations (https://card.mcmaster.ca/prevalence), and tracking of changes for each release. The website also includes a built-in BLAST instance for comparing sequences to CARD reference sequences and a web instance of RGI for resistome prediction with data visualization tools (https://card.mcmaster.ca/analyze). The download section (https://card.mcmaster.ca/download) includes CARD reference sequence data (TSV, JSON, and FASTA format), CARD Resistomes, Variants and Prevalence data (TSV, FASTA), RGI software downloads for command line usage, and all ontologies (TSV, OBO, OWL, JSON). Full documentation and open source code for the RGI is additionally available at the publicly accessible CARD GitHub (https://www.github.com/arpcard/rgi), which includes a wrapper for use with the Galaxy bioinformatics framework, a monitored issue tracker, plus instructions for using RGI via the Conda software packaging system. The ARO is additionally available through the Open Biomedical Ontologies’ OBO Foundry (http://purl.obolibrary.org/obo/aro).

The CARD biocuration and development teams are available for contact at card@mcmaster.ca and software or data releases are announced via Twitter (@arpcard) and the CARD-L mailing list (see http://arpcard.mcmaster.ca/about). In response to the 2019 European Commission's Joint Research Centre (JRC) AMR Databases Workshop, we have established the ‘AMR_Curation’ public repository for collective curation of AMR genes and mutations involving the majority of AMR database curators (e.g. NCBI, Resfinder, MEGARes, etc.) with an active and monitored curation issue tracker, a parallel AMR curation mailing list, editable Google Spreadsheet List of AMR Databases and Software, and curated Wikipedia list of AMR Databases all accessible at https://github.com/arpcard/amr_curation. We encourage researchers, software developers and AMR data curators to use this repository and associated resources to submit, discuss and resolve AMR curation issues.

## CONCLUSION

CARD has evolved substantially since our initial release (23) and previous update (24). Improvements to the ontological framework, additional annotation methods, upgraded resistome prediction software and the introduction of CARD Resistomes & Variants have all bolstered the scope of available data. We continue to expand upon the core CARD ARO with regular curation updates and public releases maintained by a growing biocuration team, while engaging in projects which use CARD for public health, clinical, agricultural and/or environmental analyses. These projects provide feedback to the CARD biocurators, further improving the AMR resources CARD provides. Similarly, CARD engages in data harmonization with other AMR resources including the NCBI National Database of Antibiotic Resistant Organisms and the Pathogen Detection Reference Gene catalog (22) and AMR research tools such as MEGARes and AMR++ (17). CARD strives to provide high-quality and carefully curated data with the goal of improving outcomes in the face of the dire AMR crisis, and looks forward to expanded collaboration among AMR databases and community engaged biocuration of AMR data.

## Supplementary Material

gkz935_Supplemental_Files

## Notes

Present address: Tammy T.Y. Lau, British Columbia Cancer Genome Sciences Centre, Vancouver, British Columbia, V5Z 1G1, Canada.

Present address: Annie A. Cheng, Stem Cell and Cancer Research Institute, McMaster University, Hamilton, Ontario, L8S 4K1, Canada.

Present address: Sihan Liu, Shift Health, Toronto, Ontario, M5R 3N5, Canada.

Present address: Anastasia Hernandez-Koutoucheva, London School of Hygiene & Tropical Medicine, University of London, London, WC1E 7HT, UK.

Present address: Arjun N. Sharma, M.G. DeGroote School of Medicine, McMaster University, Hamilton, Ontario, L8S 4K1, Canada.

Present address: Alexandra Florescu, MD/PhD Program, University of Toronto, Toronto, Ontario, M5S 1A8, Canada.

Present address: Bhavya Singh, Chemical Biology Graduate Program, McMaster University, Hamilton, Ontario, L8S 4K1, Canada.

Present address: Andrew C. Pawlowski, Wyss Institute for Biologically Inspired Engineering at Harvard University, Boston, MA 02115, USA.
