# Supplementary material for: CARD 2020: antibiotic resistome surveillance with the comprehensive antibiotic resistance database
Source: Nucleic Acids Res. 2019 Oct 29;48(D1):D517–25. doi: 10.1093/nar/gkz935 (PMC7145624; doi:10.1093/nar/gkz935)
Supplement: gkz935_Supplemental_Files [file gkz935_supplemental_files.zip › Supplementary Captions.docx]

Supplementary Figure 1**.** Example of an AMR determinant and bioinformatic detection model as found on the CARD website (v3.0.4). **A.** The ARO term “*Neisseria gonorrhoeae* gyrA conferring resistance to fluoroquinolones” incorporates a definition, its AMR Gene Family, Drug Class, and Resistance Mechanism classification tags, its ARO parent terms, and relevant peer-reviewed publications. **B.** The AMR detection model for the above term uses a protein variant model, involving a curated reference sequence, a BLASTP cut-off, and a catalogue of resistance-variant mutations that confer resistance to fluoroquinolone antibiotics.

Supplementary Table 1**.** List of pathogens included in CARD Resistomes & Variants. Pathogen names are those that appear in NCBI for the given NCBI Taxonomy accession. The number of analyzed complete chromosome and plasmid sequences, plus whole-genome shotgun assemblies, are given for each pathogen. The total number of analyzed genomes is given by pathogen and by assembly type, along with the overall total number of analyzed genomes (78,714). Note that not all analyzed assemblies contained an antibiotic resistance gene or determinant.
