# Supplementary figures and images for: CARD 2020: antibiotic resistome surveillance with the comprehensive antibiotic resistance database
Source: Nucleic Acids Res. 2019 Oct 29;48(D1):D517–25. doi: 10.1093/nar/gkz935 (PMC7145624; doi:10.1093/nar/gkz935)

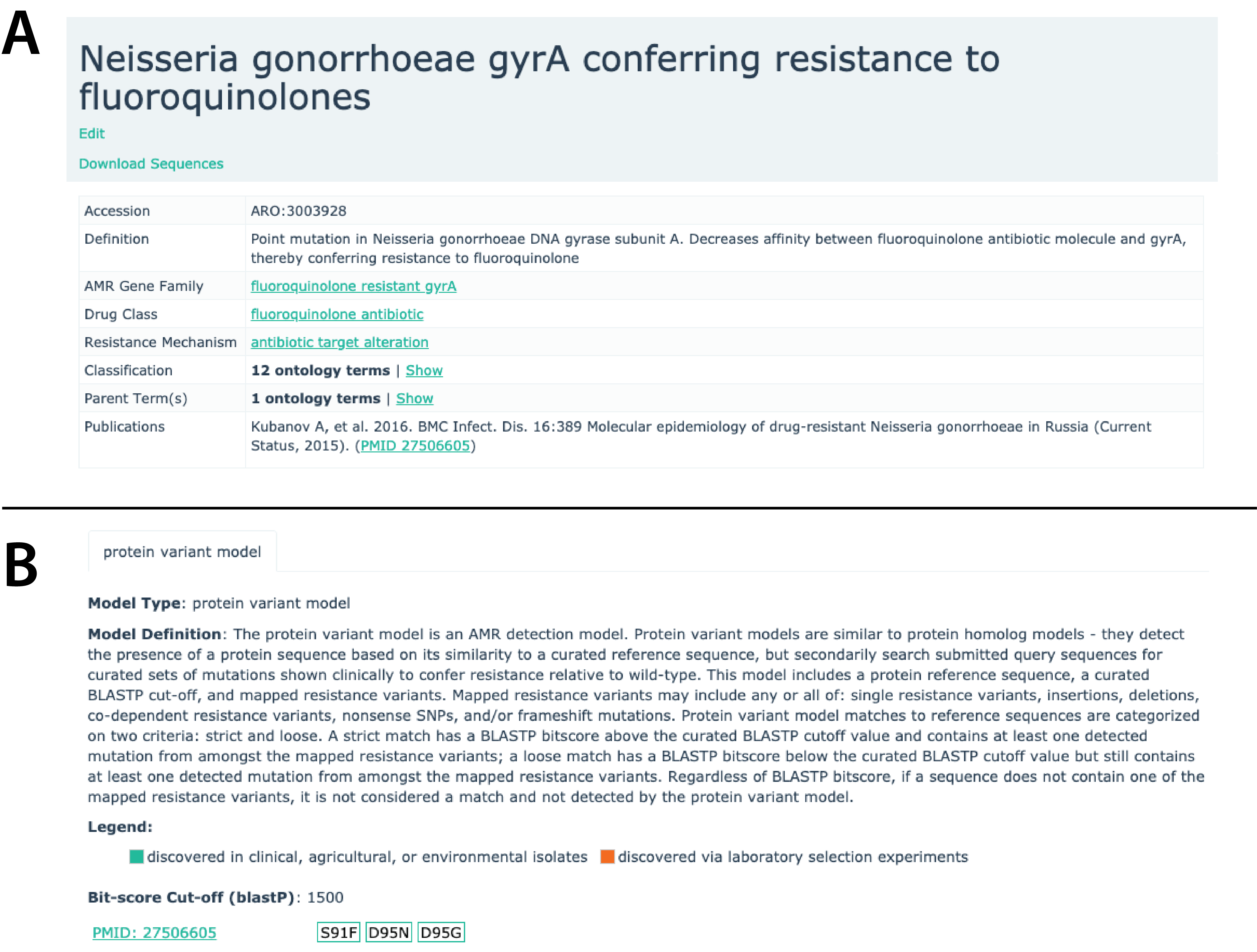

Supplement: gkz935_Supplemental_Files [file gkz935_supplemental_files.zip › Figure S1.png]
